# Impairment of Cognitive Function and Synaptic Plasticity Associated with Alteration of Information Flow in Theta and Gamma Oscillations in Melamine-Treated Rats

**DOI:** 10.1371/journal.pone.0077796

**Published:** 2013-10-30

**Authors:** Xiaxia Xu, Lei An, Xichao Mi, Tao Zhang

**Affiliations:** College of Life Sciences and Key Lab of Bioactive Materials, Ministry of Education, Nankai University, Tianjin, PR China; Centre national de la recherche scientifique, France

## Abstract

Changes of neural oscillations at a variety of physiological rhythms are effectively associated with cognitive performance. The present study investigated whether the directional indices of neural information flow (NIF) could be used to symbolize the synaptic plasticity impairment in hippocampal CA3-CA1 network in a rat model of melamine. Male Wistar rats were employed while melamine was administered at a dose of 300 mg/kg/day for 4 weeks. Behavior was measured by the Morris water maze(MWM)test. Local field potentials (LFPs) were recorded before long-term potentiation (LTP) induction. Generalized partial directed coherence (gPDC) and phase-amplitude coupling conditional mutual information (PAC_CMI) were used to measure the unidirectional indices in both theta and low gamma oscillations (LG, ∼30–50 Hz). Our results showed that melamine induced the cognition deficits consistent with the reduced LTP in CA1 area. Phase locking values (PLVs) showed that the synchronization between CA3 and CA1 in both theta and LG rhythms was reduced by melamine. In both theta and LG rhythms, unidirectional indices were significantly decreased in melamine treated rats while a similar variation trend was observed in LTP reduction, implying that the effects of melamine on cognitive impairment were possibly mediated via profound alterations of NIF on CA3-CA1 pathway in hippocampus. The results suggested that LFPs activities at these rhythms were most likely involved in determining the alterations of information flow in the hippocampal CA3-CA1 network, which might be associated with the alteration of synaptic transmission to some extent.

## Introduction

Hippocampus is known to be an essential part for learning and memory [1] with synaptic plasticity as the accepted underling mechanism [2]. Synapses are crucial for neural cell communication through the release of neurotransmitter from presynaptic neurons to postsynaptic ones. A previous study showed that the synapses in hippocampus were most chemical ones with glutamate as the primary excitatory neurotransmitter [3]. Long-term potentiation (LTP) in hippocampal CA3-CA1 pathway, which is largely mediated by glutamate neurotransmitter, is one of the important indices of synaptic plasticity [4], [5]. On the other hand, local field potential (LFP) oscillations are speculated to perform an important role in the interaction among neurons [6]. Traditionally, neuronal oscillations are classified into delta 1–4 Hz, theta 4–8 Hz, alpha 8–13 Hz, beta 13–30 Hz and gamma 30–100 Hz [7]. Theta and gamma rhythms are two prevalent rhythms in hippocampus and believed to be extremely relevant to cognition [8], [9]. It was reported that theta oscillation could mediate the glutamate synaptic information flow in the hippocampus [10], [11], which in reverse affected the activity of the hippocampal network [10], [12].

Melamine (2,4,6-triamino-5-triazine) has received extensive attentions owing to the culprit of the poison milk powder in the recent years [13], [14]. Our previous in vitro studies showed that melamine modulated the neurotransmitter release and synaptic transmission in hippocampal CA1 pyramidal neurons of infant rats [15], [16]. Moreover, melamine impaired the LTP in hippocampal CA3-CA1 pathway in rats [17], [18].

In addition, our further studies showed that there might be a close relationship between the pattern change of neural oscillations and the impairment of synaptic plasticity in either thalamocortical pathway or hippocampal CA3-CA1 pathway in the rat model of depression or vascular dementia [18]–[21]. In this study, our main aim was to examine whether the unidirectional indices of neural information flow (NIF) over theta and low gamma frequency bands (LG, ∼30–50 Hz) could be used to signify the impairment of synaptic plasticity in hippocampal CA3-CA1 pathway in melamine treated rats to some extent. Accordingly, the Morris water maze (MWM) test was performed and then spontaneous LFPs were recorded at both CA3 and CA1 regions before LTP induction in anesthetized rats. In addition,empirical mode decomposition-based phase locking value (EMD-based PLV) was applied to assess the synchronization between CA3 and CA1 regions. Moreover, modulation index (MI) was used to measure the phase-amplitude coupling in hippocampal CA3-CA1 network. Finally, the approaches of both generalized partial directed coherence (gPDC) and phase-amplitude coupling conditional mutual information (PAC_CMI) were employed to measure the unidirectional indices of NIF in and between theta and LG rhythms, in order to reveal the possible relationship between the patterns change of neural oscillation and the impairment of synaptic plasticity.

## Materials and Methods

### Animals and Treatment

Three-week-old male Wistar rats were purchased from the Laboratory Animal Center, Academy of Military Medical Science of People’s Liberation Army, and reared in the animal house in the School of Medicine in Nankai University. Animal experiments were carried out according to the protocols approved by the Committee for Animal Care at Nankai University.

Animals were randomly divided into two groups, which were melamine group (Mel, n = 6) and control group (Con, n = 6). The melamine solution (30 mg/ml) was prepared in 1% carboxymethylcellulose (CMC) solution as a suspension. It was sonicated for at least 30 min to ensure that melamine was completely dissolved. In the Mel group, rats were orally administered melamine at a dose of 300 mg/(kg day) and given once a day for 4 weeks covering day 1 to day 28. Rats in Con group only received the same dosage of 1% CMC solution [17], [18].

### Morris Water Maze Experiment

Morris water maze tests (MWM, RB-100A type, Beijing, China) were performed to monitor the rats’ ability of spatial learning and memory. The water maze consists of a tank with 150 cm in diameter and 60 cm in height. The temperature of water was maintained at about 25±1°C, while the room temperature was kept at around 28°C throughout the test. Movement of rats during test was recorded by a video tracking system (Ethovision 2.0, Noldus, Wagenigen, Netherlands) connected to a personal computer, through which signals were obtained. MWM task consisted of two phases, which were place navigation phase and spatial probe phase. In the first one, rats were subjected to ten sessions of training for five consecutive days including two sessions per day. Each session consisted of four trials. During the experiment, subsequent starting positions proceeded in a clockwise manner in the trials and animals were located in the same position on every trial at one of four starting quadrant points. The escape latency and the swimming speed were measured. If an animal failed to locate the platform within 60 s, it was placed on it for 10 s, and its escape latency was recorded as 60 s. The interval between two trials was around 5 min, and the time between two sessions was approximate 8 h. In the second phase, animals were given the probe trial test 24 hours after the place navigation phase. The platform was removed from the tank. Animals were released separately into water from the starting point and swam for 60 s as a probe test. Both quadrant dwell time and platform crossings were estimated. Only one session was tested in the phase. More details could be seen in the previous papers [17], [18].

### Electrophysiological Experiments

The signals of local field potentials were collected from both hippocampal CA1 and CA3 regions. Briefly, the animal was anesthetized by 30% urethane (4 ml/kg, i.p., Sigma-Aldrich, St. Louis, MO, U.S.A.), and then it was placed in a stereotaxic frame (Narishige, Japan). The skull was exposed and a small hole (2 mm in diameter) was drilled in its left side. Two stainless steel electrodes were slowly implanted into two sites. One was at Schaffer collaterals (4.2 mm posterior to the bregma, 3.5 mm lateral to midline and 2.5 mm ventral below the dura) from CA3 region in hippocampus. Another was at hippocampal CA1 region (3.5 mm posterior to the bregma, 2.5 mm lateral to midline and 2.0 mm ventral below the dura). Two additional electrodes, ground and reference electrodes, were placed symmetrically over the two hemispheres of the cerebellum. LFPs were fed into a multi-channel differential amplifier, and simultaneously recorded at 200 Hz sampling frequency for 5 minutes. The LFPs data are available on our website (http://www.nkbiox.com/DBWebs/eegdb/datafiles/hippocampal_EEG.rar).

After LFPs recording, long-term potentiation (LTP) was induced at the same locations, with the CA3 being the stimulating region and the CA1 the recording region. LTP were recorded at least 60 min by high-frequency stimulation. In brief, test stimuli were delivered every 30 s to evoke a response of 70% of its maximum (range 0.3–0.5 mA). After 20 min baseline, tetanic stimulation (10 pulses at 100 Hz for 2 s repeated 10 times) was used to induce the LTP. More details were illustrated in the previous papers [17], [18], [22].

### Empirical Mode Decomposition (EMD)-based Phase Locking Value (PLV)

Phase locking value is usally used to be an important index of phase synchronization, by which the degree of phase variance between two signals can be measured. Before caculating the PLVs, the phases of the two signals are extracted by the Hilbert transform. In order to obtain precise phase information, pre-digital filtering signals into narrow frequency bands is usually done. A novel approach named EMD was proposed to decompose the signal into a series of intrinsic mode functions (IMFs), which had meaningful phase definition at every time point [23]. In this way of EMD, rather than digital filter, the phase information can be extracted precisely and the signal distortion can be avoided to some extent. Following EMD, the instantaneous phases of IMFs with Hilbert transform are obtained and signed as 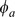 and 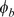. And then PLVs are determined as follows:
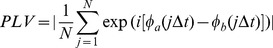

*N* stands for the length of time series and 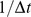 is the sampling frequency. The PLVs take values within [0 1], where 0 represents no phase synchrony and 1 the perfect phase synchrony.

### Generalized Partial Directed Coherence (gPDC) Algorithm

The algorithm is designed to determine directional influences among brain areas [24], [25]. In brief, gPDC can be considered as frequency-domain representation of Granger Causality. Here, it is based on vector autoregressive (VAR) model of the simultaneously recorded signals from both CA3 and CA1. In the gPDC algorithm, the VAR coefficients (
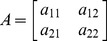
) are transformed into the frequency domain by the Fourier transform, which are 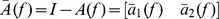.

The definition of undirectional index from CA3 to CA1 is as following,
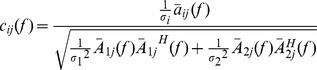


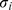
 represents the standard deviation of the model residuals. The gPDC values are in the interval [0 1], where 0 stands for the absence of an influence of CA3 on CA1 and 1 stands for CA3 is linearly predictable from CA1.

### Phase-amplitude Coupling Measured by Modulation Index (MI)

The MI measurement produces a complex valued composite signal 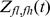 which is defined as a function of the low frequency phase 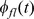 and high frequency amplitude 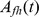: 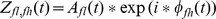.

A joint probability density function on the complex plane is created where a particular amplitude and phase value to co-occur could be identified.

The MI value is calculated as the absolute value of the average 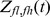


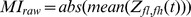



Surrogate data are generated by a time lag 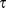 between 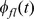 and 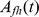:
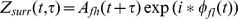



Then the normalized MI is defined as: 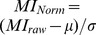



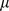
 is the mean of the surrogate lengths and 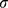 is the standard deviation.

### Phase-amplitude Directional Coupling Measured by PAC_CMI

Conditional mutual information (CMI) is a widely used directional algorithm, which is applied in the analysis of the phases of weakly coupled oscillators [26]–[28]. Simply speaking, the mutual information 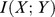 of two variables 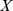 and 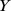 is defined as:
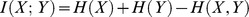



Given the variable 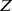, the CMI is calculated as:
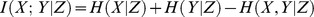



In this study, the algorithm of CMI was developed to measure the directional coupling between CA3 theta phase and CA1 LG amplitude [29]. Firstly, Hilbert transform was adopted to obtain the phase of the wide-band filtered CA3 theta rhythm (4–8 Hz) 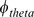 and the amplitude of narrow-band filtered CA1 gamma bands (step = 1 Hz, from 30 Hz to 60 Hz) 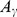. And then a second Hilbert transform was applied to gain the phase of 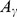 signed as 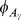. Finally, the CMI 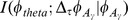 estimating the information about 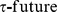 of the process 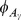 contained within 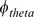 was calculated by:
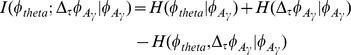



With 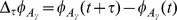


The above algorithm was defined as PAC_CMI to distinguish from the CMI used for the same-frequency directional coupling.

### Data and Statistical Analysis

All data were presented as mean ± SEM. In the MWM test, the results of escape latencies, mean swimming speed, quadrant dwell time and platform crossings were analyzed by Two-way repeated measures ANOVA. In the LTP test, the slopes of field excitatory postsynaptic potentials (fEPSPs) were expressed as the percentage change of baseline and measured by two-way ANOVA. The results of power spectrum, EMD-based PLV, gPDC, MI and PAC_CMI were measured by Mann-Whitney U test. The correlations of MI data in CA3-CA1 network were tested by Pearson correlation analysis. All the analyses were performed using SPSS 17.0 software with 0.05 as the significant level.

## Results

### Morris Water Maze (MWM) Tests

Table 1 displays the results of MWM test. Given that there were no significant differences of swimming speeds between the two groups, the detailed information was not showed in Table 1. It was found that there were gradually reduced escape latencies over trials during the five days of place navigation, indicating that the rats had learned the position of the platform in both two groups after training. However, the averaged escape latency was prolonged significantly in Mel group compared to those in the control group except for the first training day. On the sixth day, the animals have a spatial probe test. It could be seen that the remarkable reduced platform crossings (Mel: 3.25±1.58 vs. Con: 6.25±1.55, p<0.01) and the quadrant dwell time (Mel: 18.15±5.38% vs. Con: 33.88±6.84%, p<0.01) in Mel group compared to that in Con group, suggesting that the memory abilities were impaired by melamine.

**Table 1:** The results of MWM.

Group	Escape latency (s)					Platformcrossings	Quadrant dwelltime (%)
	Day 1	Day 2	Day 3	Day 4	Day 5		
Con	43.65±5.57	18.96±5.36	8.78±2.98	5.49±1.49	4.82±0.85	6.25±1.55	33.88±6.84
Mel	46.72±6.77	28.24±6.14*	16.32±5.68**	13.85±2.75**	10.70±1.84**	3.25±1.58**	18.15±5.38**

Escape latency, platform crossings and quadrant dwell time were compared between control group and melamine group, respectively. Escape latency was calculated for each day of place navigationstage, average platform crossings and quadrant dwell time were calculated for two sessions of spatial probe stage.

* P<0.05 and **p<0.01 comparison between Mel group and Con group.

### Power Spectrum Analyses

**Figure 1:**
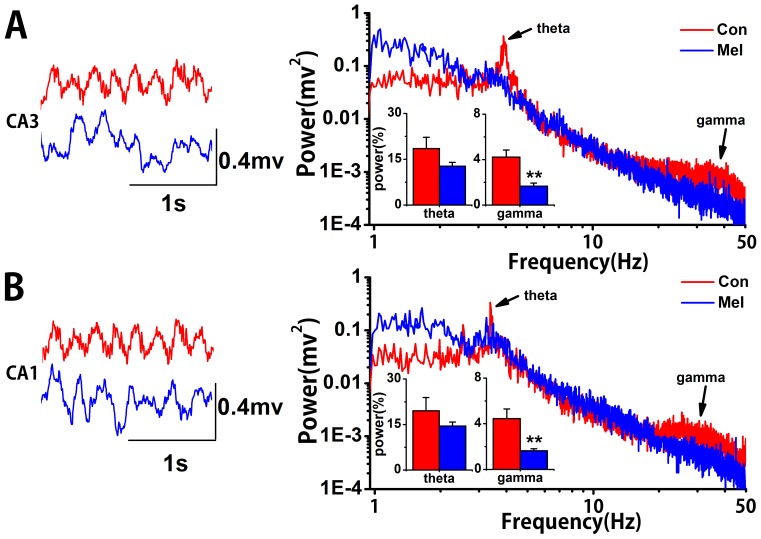
The representative LFPs and the corresponding power spectra in CA3 and CA1 regions. A. Left: The original traces of LFPs in CA3 region from a Con rat (red line) and a Mel rat (blue line). Right: The corresponding power spectra distributing from 1 Hz to 50 Hz of the two rats. Arrows present some theta and gamma rhythms. The inserts show statistical results of relative power in theta and gamma rhythms in two groups. B. Left: The original traces of LFPs in CA1 region from a Con rat (red line) and a Mel rat (blue line). Right: The corresponding power spectrum distributing from 1 Hz to 50 Hz of the two rats. Arrows present some theta and gamma rhythms. The inserts show statistical results of relative power in theta and gamma rhythms in two groups. **p<0.01 comparison between Mel group and Con group.

Fig. 1 shows an example of original traces of neural activities and the corresponding power spectral distribution plotted on a logarithmic scale in both CA1 and CA3 regions. The data were obtained from one normal rat (red line) and one melamine-treated rat (blue line). It was found that there were significant theta and gamma rhythms (indicated by arrows) in either CA3 or CA1 regions in Con group. Furthermore, there were significant increases of low frequency power in both CA3 and CA1 in melamine-treated rats compared to those in normal rats. In addition, the statistical results of the relative power spectra of theta and LG rhythms in both CA1 and CA3 regions could be seen in the inserts of the Fig. 1. It showed that there were no significant differences of relative theta power spectra between these two groups in either CA3 or CA1 regions (CA3∶12.714±1.302% vs.18.475±3.695%, U = 25, p = 0.462; CA1∶14.529±1.280% vs.19.537±4.356%,U = 25, p = 0.462). However, observable reductions of relative gamma power spectra could be seen in Mel group in both two regions (CA3∶1.665±0.275% vs. 4.228±0.633%, U = 1, p = 0.0011; CA1∶1.632±0.179% vs. 4.446±0.844%, U = 1, p = 0.0011).

### EMD-based Phase Locking Values

**Figure 2:**
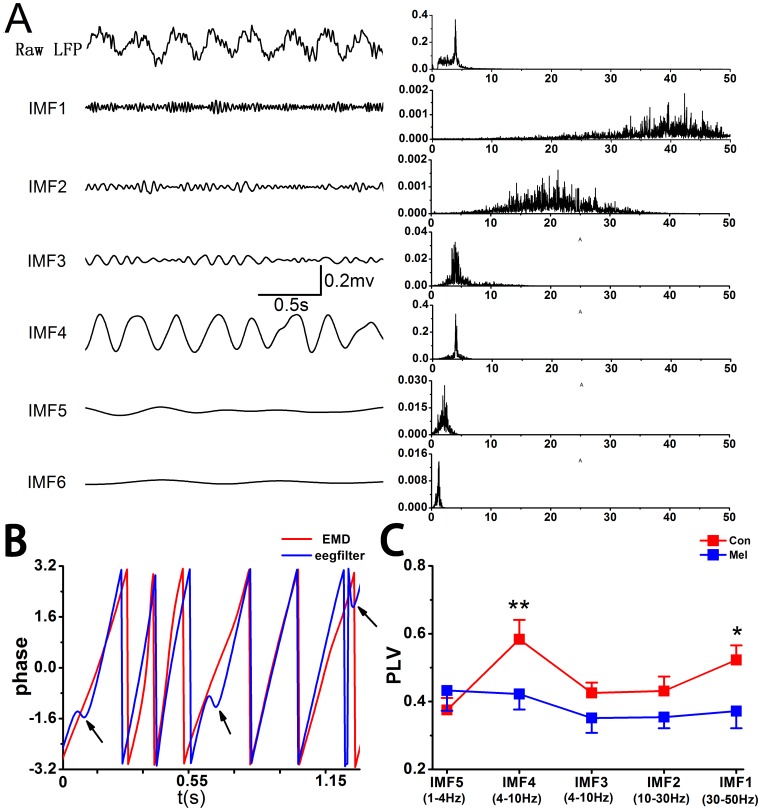
EMD-based PLV analysis between CA3 and CA1 in both Con group and Mel group. A. Examples of LFP-EMD in CA1 obtained from a normal rat. The first column illustrates the raw signals and IMFs and the second column shows the corresponding power spectra. B. The example of instantaneous phases of IMF4 (red) and band-filtered within 4 to 8 Hz (blue) signal. C. The EMD-based PLVs between CA3 and CA1 are significantly decreased in IMF4 and IMF1 in Mel group. Data represent mean ± SEM. *p<0.05 and **p<0.01 comparison between Mel group and Con group.

Fig. 2A shows an example of EMD-LFP obtained from a rat in Con group. The corresponding frequency distribution of each IMF is presented as well. It can be seen that the LFPs have been decomposed into frequency ranges corresponding well (although not strictly) with the meaningfully physiological rhythms, such as theta (IMF4) and gamma rhythms (IMF1). Fig. 2B illustrates the instantaneous phases, which are extracted by Hilbert transform of IMF4 (red curve) and filtered by digital filters (blue curve, in EEGLAB, eegfilt.m). It was found that there were visible disorders of instantaneous phases (indicated by arrows) by digital filters. Fig. 2C exhibits the strength of phase synchronization among corresponding IMFs in the two groups. In Con group, the averaged values of PLVs were above 0.5 in IMF1 and IMF4 respectively, indicating that there was fairly strong phase synchronization between CA3 and CA1 in normal state. Furthermore, it showed that the values of PLVs in IMF1 and IMF4 were significantly lower in Mel group than that in Con group (IMF1∶0.372±0.051 *vs*. 0.523±0.043, U = 13, p = 0.046; IMF4∶0.422±0.046 *vs*. 0.583±0.058, U = 10, p = 0.021).

### Directional Coupling between CA3 and CA1 at Theta and Gamma Rhythms

**Figure 3:**
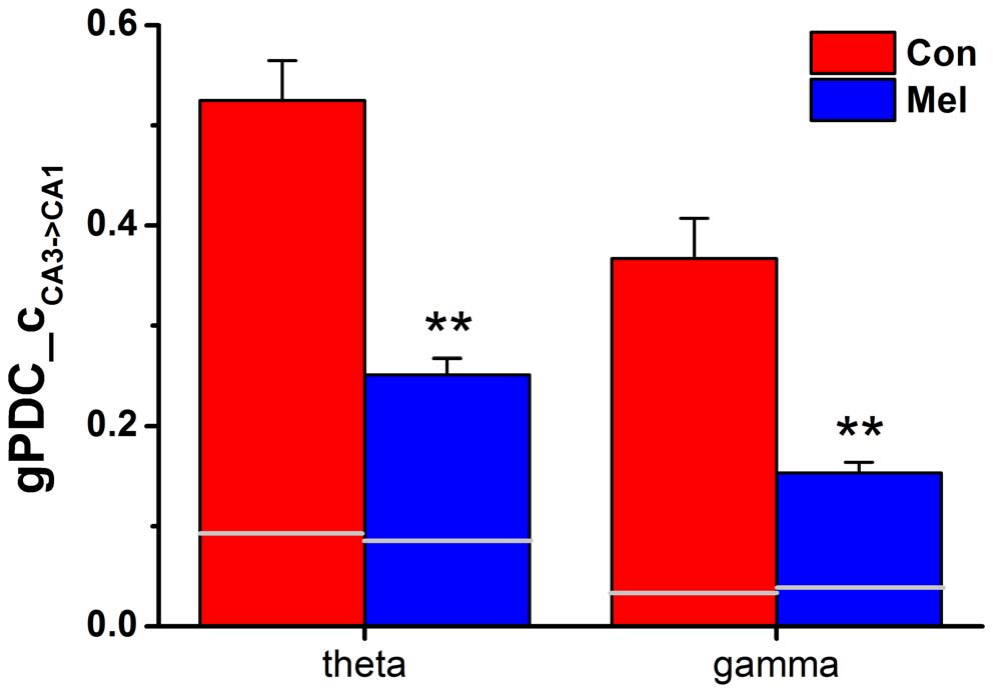
The gPDC analysis in both Con and Mel groups. The comparisons of unidirectional indices *C*_CA3->CA1_ in both theta and gamma rhythms between Con group and Mel group. The gray lines represent unidirectional indices *C*_CA1->CA3_. Data represent mean ± SEM. **P<0.01 for comparison between Con group and Mel group.

Fig. 3 shows the group data of unidirectional influence *C*_CA3→CA1_ between Con and Mel groups, while the other ones *C*_CA1→CA3_ are presented in grey lines. The unidirectional indices in both theta and LG frequency bands were significantly reduced in melamine-treated rats compared to that in normal animals (theta: 0.251±0.016 *vs*. 0.525±0.040, U = 1, p = 0.0011; gamma: 0.153±0.011 *vs*. 0.367±0.040, U = 5, p = 0.0045).

### Phase-amplitude Coupling in CA3-CA1 Network by MI

**Figure 4:**
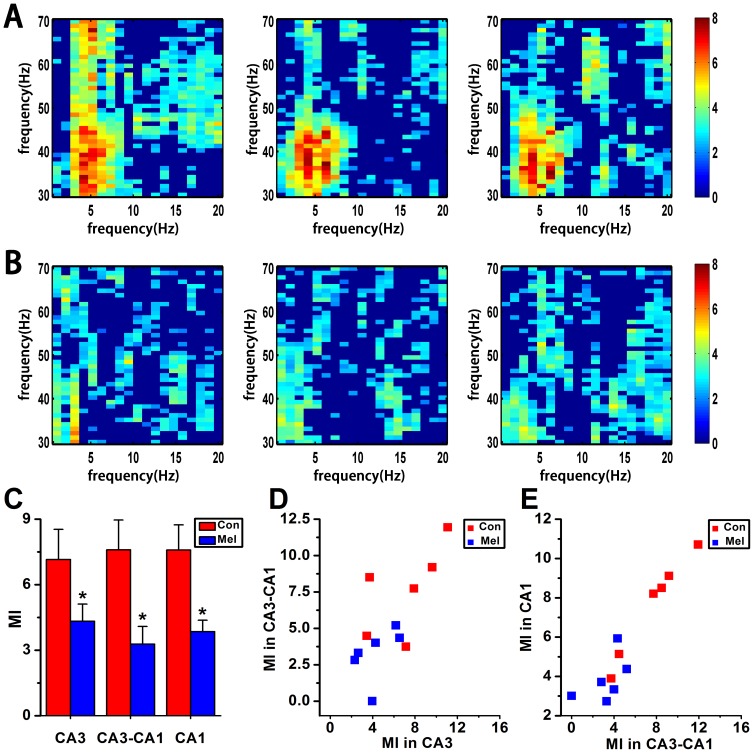
MI analysis in CA3-CA1 network in Con and Mel groups. A & B. The modulation index as a function of analytic amplitude (30 to 70 Hz) and phase (1 to 20 Hz) in CA3, CA3-CA1 and CA1 in both Con and Mel groups. The higher MI value the stronger cross frequency coupling. C. Statistical MI data of phase-amplitude coupling between theta and gamma rhythms in hippocampal CA3-CA1 network. *P<0.05 for comparison between Con group and Mel group. D. Scatter plot of CA3 MI data vs. CA3-CA1 MI data for all normal and melamine-treated rats. E. Scatter plot of CA3-CA1 MI data vs. CA1 MI data for all normal and melamine-treated rats.

In the present study, the convolution with complex Morlet wavelets of the depth 7 was used to generate analytic representations of low frequency bands (1–20 Hz, band = 1 Hz, step = 1 Hz) and high frequency bands (30–70 Hz, band = 1 Hz, step = 1 Hz). Consequently, phase components 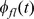 and amplitude components 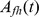 were obtained, respectively. MI measurement was performed with a window length of 40 s with 50% overlap along with the method of surrogate data. It can be seen that the theta-LG phase-amplitude modulation regularly appears at ∼4 Hz and around ∼40 Hz bins in CA3 area *per se* in Con group, which can be entrained from CA3 to CA1, and then modulate the theta-LG PAC in CA1 area *per se* (Fig. 4A). On the other hand, the coupling strength of theta-LG PAC was apparently reduced in CA3 region, and almost disappeared from CA3 to CA1 as well as in CA1 region in Mel group (Fig. 4B). There were significant differences of MI data between Con and Mel groups, respectively (Fig. 4C), in CA3 region (4.324±0.785 vs. 7.155±1.379, U = 4, p = 0.025), CA3-CA1 (3.281±0.808 vs. 7.597±1.368, U = 4, p = 0.025) and CA1 area (3.846±0.523 vs. 7.591±1.148, U = 3, p = 0.016). By comparing the normal animals and the melamine-treated ones, it was found that there were significantly positive correlations of MI values either between intra-CA3 and inter-CA3-CA1 (Fig. 4D, n = 12, r = 0.729, p = 0.007) or between inter-CA3-CA1 and intra-CA1 (Fig. 4E, n = 12, r = 0.941, p<0.0001).

### Phase-amplitude Coupling from CA3 to CA1 by PAC-CMI

**Figure 5:**
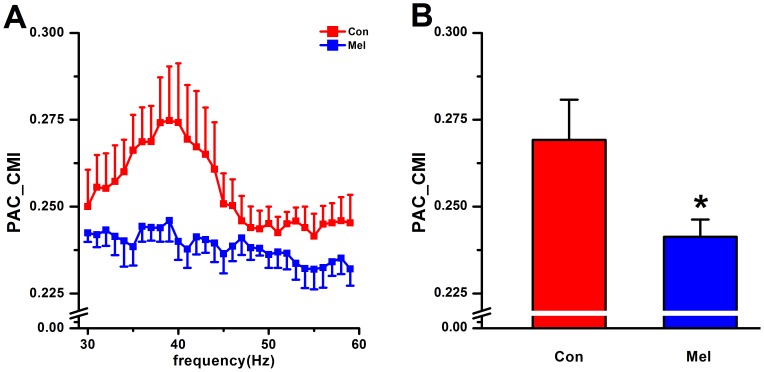
PAC-CMI analysis in CA3-CA1 network in Con and Mel groups. A. The comparison of coupling strength measured by PAC-CMI between Con and Mel groups ranged from 30 Hz to 60 Hz. B. The group data of PAC-CMI measures within the range of 30 Hz–45 Hz in Con and Mel groups. *P<0.05 for comparison between Con group and Mel group.

PAC_CMI was performed with a sliding window (length = 24 s) with 50% overlap to calculate 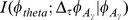, which stood for the coupling strength between the theta phase in CA3 and the phase of gamma amplitude in CA1 with a time lag 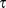
(100 ms). The data showed that there was a peak of coupling strength of theta-LG PAC around ∼40 Hz, while a relative small coupling strength existed at other parts of frequency ranges in normal animals. However, the peak of coupling strength was almost disappeared in melamine-treated rats (Fig. 5A). By Mann-Whitney U test, it can be seen that the theta-LG PAC in CA3-CA1 network is significantly decreased in Mel group compared to that in Con group (0.241±0.005 vs.0.269±0.012, U = 5, p = 0.037, Fig. 5B).

### Synaptic Plasticity

**Figure 6:**
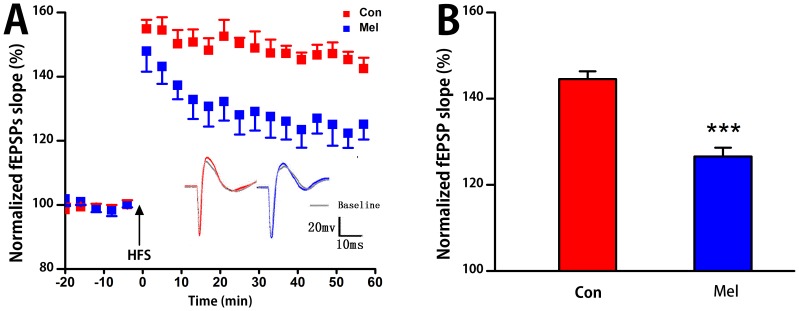
The LTP test in CA3-CA1 pathway. A. Low-frequency stimulation of Schaffer collaterals region for 20 min as the base line. The fEPSPs slope is plotted as a percentage change against the baseline before high-frequency titanic stimulation (HFS) indicated by arrows. Inset: reprehensive fEPSPs in Con group (left) and Mel group (right). B. Statistical results of fEPSPs in both Con group and Mel group. ***P<0.001 for comparison between Con group and Mel group.

The LTP results are presented in Fig. 6A. Stimulating at CA3 region evoked a basal excitatory fEPSPs in CA1. The time course of fEPSP slopes was normalized to the 20 min baseline period. High-frequency stimulation induced immediately increased fEPSP slopes, which then stabilized to a level above the baseline period for at least an hour in both two groups (Fig. 6A). The inset showed two examples of fEPSPs at baseline and high-frequency stimulation conditions of a normal rat and a melamine-treated rat. Two-way repeated measures ANOVA in LTP course for 1 h showed that there was no statistical difference of time or time× group interaction either in Con group or Mel group. Because of no statistical difference of time, Mann-Whitney U test was used to analyze the difference of mean fEPSPs slope between the two groups. It was found that there was a significant reduction in Mel group than that in Con group (126.7±1.9% *vs.* 144.5±1.7%, U = 0, p = 0.0007, Fig. 6B).

## Discussion

In the present study, the both gPDC and PAC_CMI algorithms were employed to measure the coupling strength in CA3-CA1 network either on the same frequency bands including theta and LG or cross the rhythms (theta-LG) in both normal and melamine treated rats, respectively. Along with the EMD-based PLV analysis, we aimed to show that the coupling strength between CA3 and CA1 was significantly weakened by melamine, while the comparable alterations of both LTP and cognitive functions induced by melamine were clearly observed as well. In addition, the comparison of relative theta power as well as low gamma power between normal and melamine-treated rats was also performed.

### Behavior Performance Measured by Morris Water Maze (MWM) Tests

MWM is a widely used method to examine the spatial learning and memory in animal models [30]. Our MWM results showed that with the same mean swimming speed the rats performed a significant prolongation of escape latency in Mel group compared to that in Con group in the place navigation phase. This suggested that the learning ability was significantly impaired in rats with melamine treatment. In the spatial probe period, the time spent in the target quadrant and the number of crossing target quadrant were considerably decreased in Mel group compared to that in Con group, indicating that the ability of memory was much undermined in Mel group. The above results implied that melamine could remarkably weaken the ability of the rats’ spatial learning and memory.

In the present study, it was found that the melamine rats had below chance of quadrant scores. We understand that there might be several potential reasons. Firstly, there are only a limited number of rats used in the experiment. Secondly, melamine had an effect on the reference memory, which would severely influence their performances in spatial probe phase. Thirdly, the use of clockwise rather than random choice of start points quite likely developed rats’ non-spatial strategies, such a phenomenon was occurred in a previous study [31].

### Phase Synchronization Measured by EMD-based PLV

Synchronous oscillations in physiological rhythms are usually believed as an essential mechanism linking single-neuron activity to behavior and mental disorders [11], [32]. Previous studies reported that the synchronization between CA3 and CA1 would underlie the cognitive behaviors [11], [32] with neuronal oscillation as the supposed mechanism. Furthermore, there were several investigations which were related to the appearance of specific neuronal oscillation in the given cognitive behaviors and the reduction of synchronization in particular rhythms in some impaired cognitive function [33]–[35]. It was reported that there were limitations in the estimation of phase synchronization for pre-filtering the signals into narrow frequency bands [36], [37]. Consequently, the LFP signals were decomposed by the EMD approach to obtain a series of IMFs. And each IMF had meaningful instantaneous phases at every time point. It was found that the frequency patterns were more or less corresponding to physiological rhythms, indicating the IMFs obtained by EMD were biophysically meaningful and possibly more accurate to the underlying dynamics (Fig. 2A). On the other hand, the EMD method could avoid the disorders of instantaneous phases of band-filtered signal (Fig. 2B). The EMD-based PLV analysis showed that there were significant decreases of phase locking values at both IMF4 (theta) and IMF1 (low gamma) between CA3 and CA1 in Mel group compared to that in Con group (Fig. 2C).

### Theta-gamma Nesting in Hippocampal CA3-CA1 Network

The cross-frequency phase-amplitude couplings are functional correlations, which can be modulated by cognitive task demands and might be benefit for spatiotemporal organization of cell assemblies [38]–[40] and neural coding illustrated by computational models [38], [39], [41]. In the present study, it was found that the theta rhythm (∼4 Hz) coupled with the amplitude of LG rhythm apparently in CA3 region *per se* (Fig. 4A). This phenomenon still existed in CA1 area at a more or less coordinate coupling strength. Importantly, the cross structure PAC of Con group obtained from MI measurement further discovered that the theta phase in CA3 area significantly modulated the LG amplitude in CA1 area, rather than the high gamma (>50 Hz) rhythm (Middle column of Fig. 4A). It was in accord with the report that in the hippocampus, the low gamma and high gamma oscillations were supposed to represent independent physiological processes [42]. Interestingly, the phenomenon of theta-LG nesting was almost disappeared in Mel groups (Fig. 4B & Fig. 4C), suggesting that the information entrainment across frequency bands and brain areas was significantly inhibited and led to much weak coupling in CA3-CA1 network induced by melamine. Furthermore, Pearson correlation analysis showed that there were clearly positive correlations of MI values between CA3 region and CA3-CA1 pathway (Fig. 4D), as well as between CA3-CA1 pathway and CA1 region (Fig. 4E). Consequently, these findings raise a possibility that the theta phase modulating LG amplitude in CA1 region attributes to the contribution of coupling transmission from CA3 area. A possible explanation is that the low gamma rhythm is considered to be involved in memory-retrieval mechanisms [43].

### A Possible Relationship between the NIF Changes and the Alteration of Synaptic Transmission

The above phase synchronization analysis suggested that the connection strength between hippocampal CA3 and CA1 regions was deeply weakened by melamine, which manifested that there was a disturbance in directional neural information flow in CA3-CA1 pathway. In order to evaluate the coupling directionality of NIF in hippocampal CA3-CA1 pathway, both gPDC and PAC_CMI approaches were employed. It can be seen that a more predominant driving effect occurred from CA3 to CA1 in these two groups (Fig. 3 and Fig. 5), which was in line with the anatomy synaptic projections from CA3 to CA1. Moreover, it showed that unidirectional indices from CA3 to CA1 were significantly reduced in Mel group compared to that in Con group (Fig. 3 and Fig. 5B), suggesting that melamine considerably wakened directional information transmission in hippocampal CA3-CA1 pathway. Since neural information flow through the hippocampus proceeded from dentate gyrus to CA3 and then to CA1, the unidirectional indices from CA3 to CA1 became more important to assess the alteration of NIF. Most interestingly, it was found that the alterations of the indices 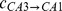, and the indices from CA3 theta to CA1 gamma were greatly consistent with the LTP alterations (Fig. 6B), suggesting that the impaired connection between hippocampal CA3 and CA1 pathway was one of the critical factors leading to the cognitive deficits. In addition, it is well known that the synapses in hippocampus are mainly excitable ones with glutamate as the major neurotransmitter. One of our previous study showed that there was a decrease of presynaptic glutamate release caused by melamine. It suggested that melamine could presynapticly reduce the release of glutamate in synaptic transmission of hippocampus, which partly resulted in damaging synaptic plasticity and further induced the impairments of cognitive functions [15], [16]. On the other hand, glutamatergic neurotransmission was essential for theta synchronization in the hippocampus [44] and this synchronization was closely linked to hippocampal LTP [45]. In consideration the above, it was inferred that the changes in glutamate system induced by melamine were the possible underline mechanisms of LTP impairments and NIF alterations in hippocampal CA3-CA1 network.

## Conclusion

In summary, the cognitive functions and CA3-CA1 synaptic plasticity in melamine treated animals were greatly impaired. Additionally, the unidirectional indices 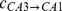 were significantly decreased in not only theta and gamma rhythms but also theta-gamma cross-frequency. It implied that the effects of melamine on cognitive impairment were possibly mediated via profound alterations of NIF on CA3-CA1 pathway in hippocampus. Consequently, the results suggested that LFPs activities at these rhythms were most likely involved in determining the alterations of information flow in the hippocampal CA3-CA1 network, which might be associated with the alteration of synaptic transmission to some extent. All the data may be helpful to further our understanding of cognitive deficits associated with neurotoxicity of melamine. However, the investigation on probing the relationship between synaptic plasticity and NIF in CA3-CA1 network associated with cognitive deficits is still at its early stage of development.
